# Dynamic summarization of bibliographic-based data

**DOI:** 10.1186/1472-6947-11-6

**Published:** 2011-02-01

**Authors:** T Elizabeth Workman, John F Hurdle

**Affiliations:** 1 Department of Biomedical Informatics, University of Utah, HSEB 5775, Salt Lake City, UT, USA

## Abstract

**Background:**

Traditional information retrieval techniques typically return excessive output when directed at large bibliographic databases. Natural Language Processing applications strive to extract salient content from the excessive data. Semantic MEDLINE, a National Library of Medicine (NLM) natural language processing application, highlights relevant information in PubMed data. However, Semantic MEDLINE implements manually coded schemas, accommodating few information needs. Currently, there are only five such schemas, while many more would be needed to realistically accommodate all potential users. The aim of this project was to develop and evaluate a statistical algorithm that automatically identifies relevant bibliographic data; the new algorithm could be incorporated into a dynamic schema to accommodate various information needs in Semantic MEDLINE, and eliminate the need for multiple schemas.

**Methods:**

We developed a flexible algorithm named Combo that combines three statistical metrics, the Kullback-Leibler Divergence (KLD), Riloff's RlogF metric (RlogF), and a new metric called PredScal, to automatically identify salient data in bibliographic text. We downloaded citations from a PubMed search query addressing the genetic etiology of bladder cancer. The citations were processed with SemRep, an NLM rule-based application that produces semantic predications. SemRep output was processed by Combo, in addition to the standard Semantic MEDLINE genetics schema and independently by the two individual KLD and RlogF metrics. We evaluated each summarization method using an existing reference standard within the task-based context of genetic database curation.

**Results:**

Combo asserted 74 genetic entities implicated in bladder cancer development, whereas the traditional schema asserted 10 genetic entities; the KLD and RlogF metrics individually asserted 77 and 69 genetic entities, respectively. Combo achieved 61% recall and 81% precision, with an F-score of 0.69. The traditional schema achieved 23% recall and 100% precision, with an F-score of 0.37. The KLD metric achieved 61% recall, 70% precision, with an F-score of 0.65. The RlogF metric achieved 61% recall, 72% precision, with an F-score of 0.66.

**Conclusions:**

Semantic MEDLINE summarization using the new Combo algorithm outperformed a conventional summarization schema in a genetic database curation task. It potentially could streamline information acquisition for other needs without having to hand-build multiple saliency schemas.

## Background

The continued growth of bibliographic databases creates challenges to users practicing traditional information retrieval (IR) techniques. Standard search techniques, when applied to large databases such as PubMed, often return large, unmanageable lists of citations that do not fulfill the searcher's information needs [1,2]. This problematic issue impedes many tasks, including secondary genetic database development. Databases such as Online Mendelian Inheritance in Man (OMIM) and Genetics Home Reference (GHR) use information from the biomedical literature to develop narrative records describing gene involvement in disease processes. Developers of secondary genetic databases built using the professional literature often rely on IR, and must invest much time and effort in procuring information [3]. The same problem prevents individuals from using IR effectively in other biomedical applications such as clinical decision support, [4] systematic review development, [5,6] and even in Google searches [7].

### NLP and Semantic MEDLINE

**Figure 1:**
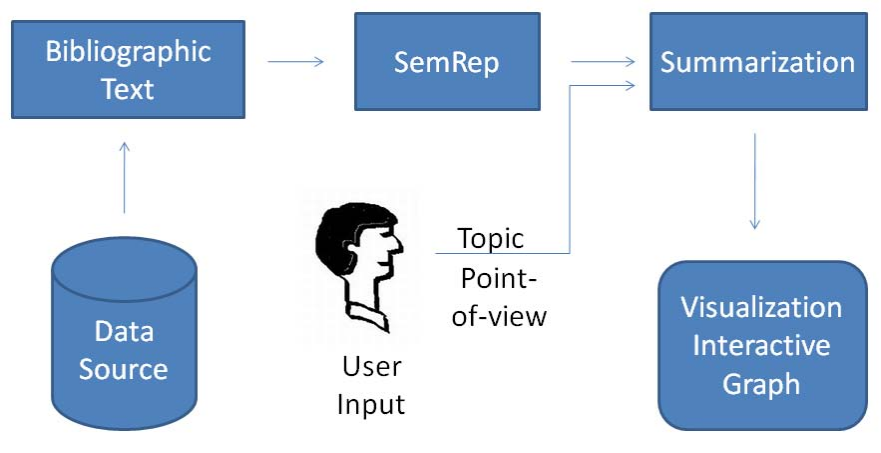
**Semantic MEDLINE**. The adaptive Combo algorithm described in this paper was designed to be incorporated into the Summarization process.

**Figure 2:**
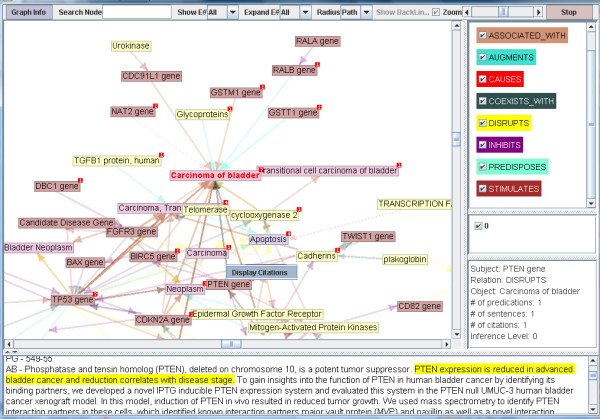
**Visualized Summarized Results**. This is an image of the Visualization process displaying summarized data addressing the genetic etiology of bladder cancer.

Natural language processing (NLP) can address this problem by identifying and summarizing text that fulfills a user's information needs in IR-procurable data. Examples of this approach include document clustering, [8] outcome polarity features in machine learning, [9] and content modeling in sentence selection [10]. NLP models leveraging transformations known as semantic predications can also address this issue. Semantic MEDLINE [11] is a multi-stage NLP system designed by researchers at the National Library of Medicine (NLM) to extract meaningful information from MEDLINE citations in the form of semantic predications, which are succinct declarations capturing the meaning of the original text. Its three core processes (Figure 1), SemRep, Summarization, and Visualization, respectively extracts semantic predications capturing the citations' content, identifies predications which are salient to a specific user-indicated information need, and displays them in a graphic representation (Figure 2). Currently, Semantic MEDLINE accommodates just a small handful of information needs, due to limitations in the Summarization stage. This problem renders Semantic MEDLINE to be an impractical tool for most users. We began this work intending to create an algorithm that would enable Semantic MEDLINE's Summarization stage to accommodate many information needs. To aid the reader in conceptualizing Semantic MEDLINE and our work to improve Summarization, we provide the following detailed description.

### SemRep

SemRep, [12] an NLM rule-based symbolic natural language processing system, extracts meaning from text in citation title and abstract fields and expresses it in the form of semantic predications. For example, if the original text reads:

"The *IGF1R *is up-regulated *in bladder cancer *compared with non-malignant bladder, and might contribute to a propensity for invasion" [13].

SemRep produces this predication:

IGF1R gene | gngm | ASSOCIATED_WITH | Carcinoma of bladder | neop

In this example, SemRep, which integrates MetaMap [14] concept mapping functionality, has determined that "IGF1R gene" and "Carcinoma of bladder" are the respective subject and object arguments in the original text by mapping the original sentence's terms to preferred concepts in the Unified Medical Language System (UMLS) [15] Metathesaurus. These arguments are connected by a predicate, in this case "ASSOCIATED_WITH," indicating the relationship that binds them in the sentence. Additionally, SemRep identifies the semantic types within the UMLS Semantic Network associated with the arguments. In this case, IGF1R is associated with the semantic type "Gene or Genome," (abbreviated as *gngm*) and Carcinoma of bladder is associated with the semantic type "Neoplastic Process" (abbreviated as *neop*).

### Summarization

Semantic MEDLINE summarization [16] filters the SemRep output, identifying the semantic predications conforming to a conceptual point-of-view, as constrained by a UMLS Metathesaurus seed topic indicated by the user. For example, a user could direct Semantic MEDLINE to summarize for the diagnosis (point-of-view) of coronary artery disease (seed topic). Summarization filters the semantic predications from the SemRep stage in four sequential steps:

Relevance: Collects semantic predications addressing the user-selected seed topic of the summary. For example, if the user chose the UMLS Metathesaurus topic "Coronary Arteriosclerosis," summarization would collect all predications that included this seed topic as a subject or object argument.

Connectivity: Augments *Relevance *semantic predications with others which share a non-seed topic argument. In continuing the example, the schema would note that the predication "Coronary Arteriosclerosis COEXISTS_WITH Inflammation" includes the argument "Inflammation." Connectivity filtering would identify other predications which also include this argument and add them to the *Relevance *group.

Novelty: Eliminates semantic predications declaring basic knowledge which users likely know, such as "Coronary Arteriosclerosis ISA Vascular Disease(s)," by paring away such predications containing general, higher level UMLS Metathesaurus concepts.

Saliency: Limits final output to semantic predications that occur most frequently. For example, the predication "tomography DIAGNOSIS Coronary Arteriosclerosis" would be included in the final output if it occurred a sufficient number of times.

When using the Semantic MEDLINE Web-based interface, users choose the desired point-of-view and seed topic from pull-down menus. Available seed topic choices are automatically determined by mapping UMLS Metathesaurus concepts to the SemRep data. Point-of-view choices are dependent on what *individually crafted *software applications known as schemas have been incorporated into Semantic MEDLINE. Within each schema, permitted semantic predications are restricted to a limited number of subject_predicate_object patterns, with semantic types serving as predicate arguments. For example, the schema designed for a *diagnosis *point of view permits only semantic predications containing CAUSES, DIAGNOSIS, LOCATION_OF, COEXISTS_WITH, PROCESS_OF, and ISA as predicates, and limits their arguments to a group of specifically named semantic types. Prior research in determining what predicates and semantic types best express a point-of-view enables schema designers to encode which specific semantic predication patterns the schema should seek.

Manually coded schema creation requires significant time and expertise. Research to determine relevant predicates and semantic types, plus time to code and test each schema, are required. At this time, there are only four schemas in place in the Semantic MEDLINE prototype Website enabling users to summarize according to four points of view: treatment of disease; [17] substance interaction; [18] diagnosis; [19] and pharmacogenomics [20]. A fifth schema summarizing data for a genetic etiology of disease point-of-view has been developed by one of us [TEW], [21] but has not yet been incorporated into the Internet version of Semantic MEDLINE. It is difficult to quantify how many points-of-view would be needed in order to satisfy most information needs; however, point-of-view refinement is roughly comparable to the conceptual scope of a subheading enhancement for MeSH subject headings, and currently there are 83 subheadings in use [22]. The five points-of-view substantially fall short when compared to subheading availability for basic IR. It would take schema developers a considerable amount of time to create enough conventional schemas to provide such summarization potential in Semantic MEDLINE.

We hypothesize that the MEDLINE output based on a user-generated PubMed query that is constrained to the desired topic and point-of-view will generate SemRep output that likely contains a semantic profile representative of the same topic and point-of-view focus. Properties of SemRep output, particularly term frequencies, may indicate the topic and point-of-view expressed in the original PubMed query. Prior efforts in leveraging term and pattern frequencies have been effective in other summarization applications [23]. An algorithm leveraging SemRep output term frequencies could dynamically infer a user's information needs and summarize accordingly. This adaptive, dynamic algorithm would accommodate summarization for diverse points of view and eliminate the need for multiple schemas.

### Project Aim

The aim of this project was to develop and evaluate an algorithm which utilizes statistical metrics to automatically identify predications salient to a seed topic and point-of-view as expressed in a PubMed search query. The work started out with the initial task of supporting secondary genetic database curation, but the method is general enough to apply to other tasks. The use of SemRep as a semantic predication generator is a choice of convenience. As long as there is a sufficiently representative collection of texts available for the algorithm to use in appraising data, the methods described here are sufficiently generalizable to apply to semantic predications produced by other applications [24,25].

## Methods

We developed a new algorithm that dynamically identifies salient SemRep output, and then evaluated its utility by comparing its performance to that of a conventional summarization schema, as well as two of the individual metrics which form the algorithm. MEDLINE data was harvested via PubMed using a query expressing a specific topic and point-of-view. The citations were processed by SemRep. We summarized the SemRep output by applying the new algorithm, guided by the four-filter architecture described earlier. To assess the algorithm's collective efficiency, we also summarized the SemRep data by separately applying the algorithm's two core metrics. We also processed the SemRep output with a conventional summarization schema designed to filter data according to a genetic etiology of disease point-of-view. Bladder cancer served as the seed topic in each case. In order to evaluate outputs, we simulated the task of secondary genetic database curation. In this task, a semantic predication such as "TP53 gene | ASSOCIATED_WITH | Carcinoma of bladder" is desirable, because it offers data salient to the work of annotating gene and disease process information in a database like OMIM or GHR. The semantic predicate "Excision | TREATS | Carcinoma of bladder" is not desirable, because it offers no information addressing gene function in disease development for database curators. We extracted genetic entities (e.g., genes, proteins) from the outputs of all summarizing methods. Using a reference standard, we measured precision, recall, and F-scores for the outputs of all summarization methods.

### Algorithm Development

After researching several different approaches, adaptations of the Kullback-Leibler Divergence [26] and Riloff''s RlogF metric [27,28] demonstrated promising capabilities in identifying salient predications in SemRep output.

#### Kullback-Leibler Divergence

The Kullback-Leibler Divergence (KLD) determines the difference between a true distribution (P) and an assumed distribution (Q):

$$\text{D}\left(\text{P}||\text{Q}\right)=\sum \text{ P}\left(\text{x}\right){\text{log}}_{\text{2}}\left(\text{P}\left(\text{x}\right)/\text{Q}\left(\text{x}\right)\right)$$

where x represents the relative frequency of each unique predicate in each distribution. In our case, we compare the distribution of SemRep predicates resulting from a PubMed query that expresses a seed topic and a point-of-view, as distribution P, with a large dataset of predicates expressing the seed topic from all of MEDLINE (i.e., representing all points of view), as distribution Q. We compare only shared predicates. The individual KLD calculation (before summing) assigns a value to each predicate indicating its prevalence in distribution P, expressing the single point-of-view. For example, if the predicate ASSOCIATED_WITH had a relative frequency of 0.290 in distribution P and 0.076 in distribution Q, its KLD value would be 0.5603. Semantic predications in both distributions are limited to those containing a chosen UMLS Metathesaurus seed topic before KLD analysis. A database that contains SemRep output for all MEDLINE citations published between Jan 1, 1999 to August 31, 2009 served as the data source for distribution Q.

#### RlogF

The RlogF metric was designed to assess the relevance of extracted patterns in unlabeled text, and was applied to SemRep output to measure the significance of semantic types as bound to a single predicate. Because the semantic type associated with the seed topic is so prevalent in the data, RlogF was adapted to assess the significance of a *non-seed topic semantic type *as bound to a predicate in each semantic predication:

$$\text{RlogF}\left({\text{pattern}}_{\text{i}}\right)={\text{log}}_{\text{2}}\left({\text{semantic type frequency}}_{\text{i}}\right)*\text{P}\left(\text{relevant}|{\text{pattern}}_{\text{i}}\right)$$

The conditional probability (P(relevant | pattern_i_)) is the quotient of the raw frequency of a specific semantic type as bound to a given predicate, divided by the raw frequency of all semantic types as bound to the same predicate:

$$\text{P}\left(\text{relevant}|{\text{pattern}}_{\text{i}}\right)=\frac{{\text{semantic type frequency}}_{\text{i}}}{{\text{total frequency}}_{\text{i}}}$$

The pattern's conditional probability is weighted by the log of its frequency, represented here as log_2_(semantic type frequency_i_). For example, if the non-seed topic semantic type *gngm *occurs with the predicate ASSOCIATED_WITH 107 times, and all combined non-seed semantic types occur with the same predicate 171 times, the resulting RlogF score will be 4.22. The use of the log_2_() term serves to flatten the dynamic range of the probability space, rewarding semantic types that are very strongly correlated with the relevant patterns while still rewarding moderately correlated types that occur very frequently.

#### PredScal

Raw RlogF scores can exceed raw KLD scores, yet they express a different relationship in SemRep's output space. KLD scores express a proportional relationship among predicates across the entire dataset, while RlogF values express a binding between a single predicate and its associated semantic types. Therefore, we created a scaling function named PredScal to scale RlogF values according to the spatial proportions of predicates in a given dataset:

$$\text{PredScal}=\text{1}/{\text{log}}_{\text{2}}\left(\text{c}\right)$$

In this metric, c represents the count of unique predicates in a dataset. For example, if there were 16 unique predicates in a dataset, PredScal would equal a scaling factor of 0.25.

The three metrics were combined to form a new algorithm, called "Combo," to evaluate SemRep data:

Combo=(RlogF*PredScal)*KLD

Each semantic predication has the form SemanticType_a _*predicate_i _*SemanticType_b_, so its Combo score is calculated by scaling the RlogF metric of the predicate/non-seed topic semantic type with the PredScal metric, then multiplying the result with the predicate's KLD score.

### Data

MEDLINE citations returned by PubMed for the following search query were downloaded:

("2003/01/01"[Publication Date]: "2008/07/31"[Publication Date]) AND (Urinary Bladder Neoplasms/genetics[majr] AND Urinary Bladder Neoplasms/etiology[majr]) AND English[la]

The search query focuses on the genetic etiology (the point-of-view) of bladder cancer (the topic). In this query, we limited citation output to a five-year span merely as a convenience, allowing us to utilize an existing reference standard.

### Algorithm Application

We utilized Combo as the operative mechanism in the final *Saliency *filter in the four-filter architecture. To obtain results for the *Relevancy *filter, we extracted all novel semantic predications from the SemRep data which included the UMLS Metathesaurus seed topic "Carcinoma of bladder" as an argument. Then we applied the Combo algorithm to derive a score for each semantic predication. The semantic type associated with "Carcinoma of bladder" is *neop*; the non-seed semantic type associated with the opposing subject/object argument in each semantic predication was used in performing the algorithm's RlogF calculation.

In order to explore salient predications that would result from the *Connectivity *filter, we performed a similar analysis on the novel SemRep output which did not include the UMLS Metathesaurus seed topic "Carcinoma of bladder," but did share the non-seed topic semantic type gngm with two of the highly ranked *Relevancy *predications. We also applied the Combo algorithm to derive a score for each semantic predication in this *Connectivity *group. We calculated the RlogF scores using the semantic type other than the seed gngm in deriving a Combo score for each semantic predication. In the case of these predications sharing the gngm semantic type, if their other semantic type was *neop *it was associated with UMLS Metathesaurus concepts such as "Neoplasm progression" and "Carcinoma, Transitional Cell."

To reiterate the four-filter architecture application description, we note that in both of the above procedures (i.e., *Relevance *and *Connectivity *filtering) we included only novel predications in our analyses, thus simulating *Novelty *filtering. The *Saliency *filtering phase consisted of the Combo algorithm application to identify the most informative predications.

To serve our task-based analysis, we extracted all genetic entities noted as arguments in the four top-ranked novel *Relevancy *semantic predication patterns, and the top-ranked novel *Connectivity *pattern (see Table 1).

**Table 1:** Combo Scores of Top-Ranking Patterns in Novel Relevance and Novel Connectivity Analyses; non-seed semantic types are indicated in square brackets.

Relevancy Analysis Seed Topic: Carcinoma of bladder	Combo Score
[gngm] ASSOCIATED_WITH neop	0.592531

[gngm] PREDISPOSES neop	0.205778

[aapp] ASSOCIATED_WITH neop	0.152883

[aapp] PREDISPOSES neop	0.039868

**Connectivity Analysis Shared Semantic Type: gngm**	**Combo Score**

gngm ASSOCIATED_WITH [neop]	0.873016

### Individual Metric Application

To assess the efficiency of the combined metrics in the Combo algorithm, we also separately applied the KLD and RlogF metrics in summarizing the SemRep data within the four-filter architecture. To simulate the *Relevance *stage for KLD summarization, we identified the four predicates with the highest KLD scores which included the seed topic "Carcinoma of bladder" as a subject or object argument. All novel semantic predications including these predicates and the seed topic were extracted as salient output. To simulate the *Connectivity *stage, we identified the highest scoring predicate, using the most prominent shared semantic type argument from the *Relevance *stage as the shared argument seed in KLD computation. All novel semantic predications containing the top *Connectivity *stage predicate and shared semantic type were also extracted as salient output. We extracted all genetic entities serving as subject or object arguments in the salient output.

To independently apply the RlogF metric in summarizing the SemRep output within the *Relevance *stage, we identified the four top scoring RlogF predicate/non-seed semantic type pairings among all semantic predications which included the seed topic "Carcinoma of bladder". Novel semantic predications which included these top four predicate/non-seed semantic type pairs were extracted as salient output. To simulate the *Connectivity *summarization phase, we identified the predicate/non-seed semantic type pair with the highest RlogF score among all semantic predications that contained the most prominent shared semantic type from the *Relevance *phase. Novel semantic predications containing this predicate/non-seed semantic type pair were also set aside as salient output. We extracted all genetic entities serving as subject or object arguments in the salient output.

### Conventional Schema

A conventional schema designed to summarize for the point-of-view of genetic etiology of disease also processed the SemRep data. Genetic entities serving as arguments were also extracted from its output.

### Evaluation

To evaluate the four groups of extracted genetic entities, we normalized their names to coincide with the associated gene names in Entrez Gene, and compared them to a reference standard of genes implicated in bladder cancer development in selected OMIM and GHR records, originating from primary literature published between January 1, 2003 and July 31, 2008. To normalize protein, peptide, and amino acid terms, we identified the gene which exclusively produced the entity according to Entrez Gene records, and replaced each term with the matching gene name. Terms which were too general to be matched to a specific gene were discarded. The reference standard had been assembled prior to this study in order to evaluate the conventional schema [21]. One of us (TEW) and another colleague reviewed OMIM and GHR records having a major focus on bladder cancer and the genes potentially involved in its development. They identified 13 genes which had proven secondary genetic database curation appeal because of their descriptions in the OMIM and GHR records. Results for this study were evaluated in terms of recall, precision, and F-score.

## Results

The base search query provided 667 citations focused on genetic etiology of bladder cancer. Leveraging MeSH indexing (i.e., the use of the *[majr] *flag in the query above) resulted in citations that included both the genetic and the etiologic factors of bladder cancer as major themes. SemRep processed the 667 citations, resulting in 5,421 semantic predications.

The four summarization methods provided diverse results in terms of raw and task-based output. The Combo summarization method identified 201 salient semantic predications, while the KLD metric alone identified 630 salient semantic predications, and the RlogF metric alone identified 177 salient semantic predications. The conventional schema identified 112 salient semantic predications. The top-ranking novel *Relevance *and *Connectivity *predication scores from the Combo, KLD, and RlogF analyses are listed in Tables 1, 2 and 3. There were 74 individual genes identified as implicated in bladder cancer development in the Combo output. The KLD metric alone identified 77 genes, and the RlogF metric alone identified 69 genes implicated in bladder cancer development. The conventional schema output included 10 such implicated genes.

**Table 2:** Kullback-Leibler Divergence Scores of Top-Ranking Predicates in Novel Relevance and Novel Connectivity Analysis

Relevance Analysis Seed Topic: Carcinoma of bladder	KLD Score
ASSOCIATED_WITH	0.561861059

PREDISPOSES	0.299181776

AFFECTS	0.088951936

PART_OF	0.034851914

**Connectivity Analysis Shared Semantic Type: gngm**	

ASSOCIATED_WITH	0.5553145

**Table 3:** RlogF Scores of Top-Ranking Predicate/Non-seed Semantic Type Pairs in Novel Relevance and Novel Connectivity Analysis.

Relevance Analysis Seed Topic: Carcinoma of bladder	RlogF Score
gngm ASSOCIATED_WITH	4.218344839

topp TREATS	2.96127605

ISA neop	2.807354922

gngm PREDISPOSES	2.751207824

**Connectivity Analysis Shared Semantic Type: gngm**	

ASSOCIATED_WITH neop	7.208071323

Recall for the four summarization methods was calculated by comparing outputs to the reference standard of genes noted in relevant GHR and OMIM records as noteworthy in bladder cancer development. Summarization using the Combo algorithm achieved 61% recall. The KLD and Rlogf summarization methods also achieved 61% recall. The conventional schema achieved 23% recall. The reference standard includes genes implicated in bladder cancer development in specific GHR and OMIM records, but likely does not represent a comprehensive list of genes associated with bladder cancer development. The reference standard provides a list of genes whose value has already been confirmed within the task of secondary genetic database curation, because GHR and OMIM curators have annotated their potential roles in bladder cancer development. The results of the reference standard analysis are listed in Table 4.

**Table 4:** Recall Results with Reference Standard (TP = True Positive; FN = False Negative)

Gene	Combo Analysis	KLD Analysis	RlogF Analysis	Conventional Schema
FGFR3	TP	TP	TP	TP

XPD	TP	TP	TP	FN

RAG1	FN	FN	FN	FN

TP53	TP	TP	TP	TP

MTCYB	FN	FN	FN	FN

HRAS	TP	TP	TP	FN

NAT2	TP	TP	TP	TP

RB1	TP	TP	TP	FN

TSC1	TP	TP	TP	FN

ATM	FN	FN	FN	FN

TGFB1	FN	FN	FN	FN

MDM2	TP	TP	TP	FN

ERBB3	FN	FN	FN	FN

Recall	61%	61%	61%	23%

Precision was evaluated by taking the previously established true positive findings into account with the additional genes included as arguments in the semantic predications identified as salient by the four summarization methods. To assess validity (true positive or false positive status) for the additional genes, Genes into Reference (GeneRIF) notations in relevant Entrez Gene records were reviewed for disease process implication, thus confirming appeal for the simulated task of genetic database curation. If the relevant Entrez Gene record did not contain applicable GeneRIFs, but otherwise noted bladder cancer association, the gene was assigned true positive status. Summarization with the new Combo algorithm achieved 81% precision. The KLD summarization method attained 70% precision, and the RlogF method achieved 72% precision. The conventional schema attained 100% precision. Table 5 highlights precision scores.

**Table 5:** Precision Results (TP = True Positive; FP = False Positive)

	Combo Analysis	KLD Analysis	RlogF Analysis	Conventional Schema
TP	60	54	50	10

FP	14	23	19	0

Total	74	77	69	10

Precision	81%	70%	72%	100%

We calculated F-scores for each method to assess a balance between recall and precision. The Combo summarization method resulted in an F-score of 0.69. The KLD and RlogF methods yielded F-scores of 0.65 and 0.66, respectively. Summarization with the conventional schema produced an F-score of 0.37.

## Discussion

In this study's task-based context (i.e., genetic database curation), summarization with the new Combo algorithm outperformed the conventional schema in terms of raw output and recall, while maintaining reasonable precision. Combo also produced a higher F-score than the separate KLD and RlogF applications, thus attaining a slightly superior balance of recall and precision. All of the five patterns that Combo identified as salient (Table 1) yielded semantic predications containing gene arguments, with an average of 26 separate arguments per pattern. In the separate KLD application, the predicates AFFECTS and PART OF, when paired with the seed topic in the *Relevance *phase, together produced only nine gene arguments while all salient KLD patterns (Table 2) produced an average of 32 separate arguments. The nine arguments produced by AFFECTS and PART_OF were duplicated elsewhere in the KLD analysis. Each RlogF pattern (Table 3) produced an average of 22 separate gene arguments. Semantic predications matching the two RlogF salient patterns *Therapeutic or Preventive Procedure TREATS *(topp TREATS) and *ISA Neoplasm *(ISA neop) in *Relevance *summarization did not have gene arguments, and were therefore unproductive. The Combo, KLD, and RlogF applications performed identically in terms of recall; each method produced the same genes from the reference standard. Combo outperformed the separate KLD and RlogF applications in terms of precision. It produced more genes with validated curation potential.

Because the Combo algorithm is designed to adaptively identify relevant data through analysis of a SemRep dataset's individual properties, its generalizability gives it potential to address information needs other than genetic disease etiology. The algorithm could be encoded into a very flexible schema for integration into the Semantic MEDLINE model. The new dynamic schema could potentially enable Semantic MEDLINE to summarize for many points of view, thus transforming it into a dynamic NLP application for a diverse range of needs. The Visualization component in Semantic MEDLINE would provide a graphical representation of the summarized results (see an example of visualized genetic etiology of bladder cancer findings in Figure 2).

There are several information needs that a dynamic schema could address. Secondary database curators could implement it in order to find additional genes associated with a disease process, as recorded in bibliographic text. Researchers in other fields may also benefit from dynamic text summarization. The following vignettes illustrate Combo's generalizability by exploring how Semantic MEDLINE, empowered by this new algorithm, may benefit multiple information needs.

### Primary Research

In the initial work of research, scientists usually review prior studies related to a planned investigation. This can be a time-consuming step. For example, scientists exploring the causes of myocardial infarction in humans must review over 17,000 major studies found in PubMed. Semantic MEDLINE with the Combo algorithm could facilitate this type of data appraisal. For example, researchers could execute the following query:

myocardial infarction/etiology[Majr] Limits: Humans

Then, they could choose the UMLS Metathesaurus seed topic(s) addressing their needs. Results would then be reviewed using the graphic display, giving an immediate overview of salient content. The researchers could execute searches limited by time ranges (e.g., items published within a three year period) to simplify the amount of data within the Visualization graph, and to note how research chronologically evolved in the field. Effective use of Semantic MEDLINE as a research appraisal tool could accelerate investigational studies and eventually quicken the bench to bedside process in clinical care.

### Clinical Decision Support

Online biomedical databases such as MEDLINE can answer clinicians' questions, but are time-consuming to use [29]. Semantic MEDLINE with the Combo algorithm could quickly summarize large amounts of citations and provide a graphic representation of data addressing many information needs. Consider the following scenario: a physician assistant (P.A.) wants to prevent future injury to an elderly patient experiencing recurrent hip fractures. The P.A. submits the search "Hip Fractures[mesh] AND recurrent" and then chooses "Hip fractures" as the UMLS Metathesaurus seed topic. Using the graphic display, the P.A. notes that dementia [30] is associated with recurrent hip fracture. The P.A. realizes that addressing this comorbidity may prevent future fractures. Sorting through the citations by hand would have required too much time to be practical; acquiring the information using Semantic MEDLINE takes less than a minute.

### Systematic Reviews

Evidence-Based Medicine (EBM) is "the conscientious, explicit, and judicious use of current best evidence in making decisions about the care of individual patients" [31]. Research based on EBM principles provides scientifically grounded information for patient care. Consider the following scenario: a working group within the Cochrane Collaboration wishes to update a review offering dietary advice for cardiovascular disease reduction [32]. They compose and execute the following PubMed query:

diet[mesh] AND cardiovascular diseases/prevention and control[mesh]

The search is limited to Randomized Controlled Trials, which results in 432 citations.

The group uses Semantic MEDLINE with the Combo algorithm to assess the citations, choosing the most relevant seed topics. This provides them with an immediate visual assessment of the randomized controlled trials, giving them a starting point in evaluating and selecting research to include in their systematic review.

## Limitations

This study compared conventional schema output to the statistical algorithm's performance in the context of the single task of secondary genetic database curation for the genetic etiology of bladder cancer. We cannot quantify its performance in other applications until similar research determines it. However, Semantic MEDLINE with conventional summarization has proven to be effective in identifying evidence-based treatment of 50 diseases [17]. Considering the overall performance improvement demonstrated by the new statistical algorithm over traditional summarization, it also holds promise in other applications. In conducting this study, we did not have access to curators' individual protocol and thought processes, which are clearly essential to know in a real-world curation application of Combo. We can, however, speculate on what information is valuable in database curation by what is noted in the biomedical literature. We should also note that Summarization performance in the Semantic MEDLINE model is dependent on the query results, specifically, the search query's performance, the quality of the citations garnered in IR, and SemRep's accuracy in capturing the citations' content.

## Conclusion

In this paper we described the development of a statistically based algorithm known as Combo that automatically summarizes SemRep semantic predications for a topic and a point-of-view in the Semantic MEDLINE model. We evaluated summarization utilizing Combo by comparing it to conventional summarization, using a previously established reference standard, in the task-based context of secondary genetic database curation. We also proposed real-world scenarios showing how Semantic MEDLINE, empowered with the new Combo algorithm, could benefit additional information needs. Combo is not limited to predications generated by SemRep; any predication generator that produces subject_predicate_object triplets could benefit from Combo.

## Abbreviations

IR: Information Retrieval; NLM: National Library of Medicine; NLP: Natural Language Processing; GHR: Genetics Home Reference; OMIM: Online Mendelian Inheritance in Man; P.A.: Physician Assistant; UMLS: Unified Medical Language System.

## Competing interests

The authors declare that they have no competing interests.

## Authors' contributions

TEW developed the Combo algorithm, designed the study, harvested the data, oversaw data processing with SemRep and the conventional genetics schema, performed the Combo, KLD, and RlogF summarizations, conducted the evaluation, and wrote the original manuscript. JFH provided essential manuscript revisions, project supervision, and mentoring, and suggested the use of the RlogF metric. Both authors read and approved the final manuscript.

## Pre-publication history

The pre-publication history for this paper can be accessed here:

http://www.biomedcentral.com/1472-6947/11/6/prepub
